# ASIC1A in the bed nucleus of the stria terminalis mediates TMT-evoked freezing

**DOI:** 10.3389/fnins.2015.00239

**Published:** 2015-07-21

**Authors:** Rebecca J. Taugher, Ali Ghobbeh, Levi P. Sowers, Rong Fan, John A. Wemmie

**Affiliations:** ^1^Department of Psychiatry, University of IowaIowa City, IA, USA; ^2^Department of Veterans Affairs Medical CenterIowa City, IA, USA; ^3^Department of Molecular Physiology and Biophysics, University of IowaIowa City, IA, USA

**Keywords:** TMT, bed nucleus of the stria terminalis, ASIC1A, respiratory rate, predator odor

## Abstract

Mice display an unconditioned freezing response to TMT, a predator odor isolated from fox feces. Here we found that in addition to freezing, TMT caused mice to decrease breathing rate, perhaps because of the aversive smell. Consistent with this possibility, olfactory bulb lesions attenuated this effect of TMT, as well as freezing. Interestingly, butyric acid, another foul odor, also caused mice to reduce breathing rate. However, unlike TMT, butyric acid did not induce freezing. Thus, although these aversive odors may affect breathing, the unpleasant smell and suppression of breathing by themselves are insufficient to cause freezing. Because the acid-sensing ion channel-1A (ASIC1A) has been previously implicated in TMT-evoked freezing, we tested whether *Asic1a* disruption also altered breathing. We found that TMT reduced breathing rate in both *Asic1a^+/+^* and *Asic1a^−/−^* mice, suggesting that ASIC1A is not required for TMT to inhibit breathing and that the absence of TMT-evoked freezing in the *Asic1a^−/−^* mice is not due to an inability to detect TMT. These observations further indicate that ASIC1A must affect TMT freezing in another way. Because the bed nucleus of the stria terminalis (BNST) has been critically implicated in TMT-evoked freezing and robustly expresses ASIC1A, we tested whether ASIC1A in the BNST plays a role in TMT-evoked freezing. We disrupted ASIC1A in the BNST of *Asic1a^loxP/loxP^* mice by delivering Cre recombinase to the BNST with an adeno-associated virus (AAV) vector. We found that disrupting ASIC1A in the BNST reduced TMT-evoked freezing relative to control mice in which a virus expressing eGFP was injected. To test whether ASIC1A in the BNST was sufficient to increase TMT-evoked freezing, we used another AAV vector to express ASIC1A in the BNST of *Asic1a^−/−^* mice. We found region-restricted expression of ASIC1A in the BNST increased TMT-elicited freezing. Together, these data suggest that the BNST is a key site of ASIC1A action in TMT-evoked freezing.

## Introduction

Exposure to odors from predators, such as foxes and cats, can elicit unconditioned freezing and avoidance responses in rodents (Takahashi et al., 2005). Because of the lack of a clear conditioning event, these predator odor-evoked responses are thought to be analogous to specific phobias in humans (Rosen et al., 2008).

One widely used odor is trimethylthiazoline (TMT), a synthetic compound originally isolated from fox feces (Fendt et al., 2005). TMT evokes freezing and avoidance responses in rodents (Vernet-Maury et al., 1984; Fendt et al., 2003). Because this freezing is seen even in naïve animals raised in the laboratory setting, TMT is thought to be an unconditioned fear-evoking stimulus. Though the neurocircuitry of TMT-evoked freezing has not been completely delineated, growing evidence suggests that it relies on different circuitry than conditioned fear behaviors. Lesion studies indicate a critical role for the basolateral, but not the medial amygdala in fear conditioning (Ledoux et al., 1990; Nader et al., 2001); conversely, lesion studies point to the medial amygdala playing a critical role in TMT-evoked freezing and suggest that the basolateral amygdala plays a smaller, modulatory role (Wallace and Rosen, 2001; Fendt et al., 2003; Muller and Fendt, 2006). The bed nucleus of the stria terminalis (BNST) has also been firmly implicated in TMT-evoked freezing. Exposure to TMT induces robust c-Fos activity in the BNST (Day et al., 2004; Asok et al., 2013), and inactivation of the BNST blocks TMT-evoked freezing (Fendt et al., 2003).

Our recent studies suggest that the BNST, in addition to its role in TMT-evoked freezing, plays a key role in unconditioned freezing and avoidance responses to carbon dioxide (CO_2_) (Taugher et al., 2014). In addition, we found that a receptor in the BNST that is activated by extracellular acidosis, the acid-sensing ion channel-1a (ASIC1A), was both necessary and sufficient for CO_2_-evoked freezing (Taugher et al., 2014). Curiously, this same receptor has also been implicated in TMT-evoked freezing: global disruption of *Asic1a* attenuated freezing to TMT as well as freezing to CO_2_ (Coryell et al., 2007, 2008; Ziemann et al., 2009; Price et al., 2014; Taugher et al., 2014). Thus, we hypothesized that the BNST might be a critical site of ASIC1A action in TMT-evoked freezing.

## Materials and methods

### Mice

*Asic1a^−/−^* and *Asic1a^loxP/+^* mice were generated as previously described (Wemmie et al., 2002; Kreple et al., 2014; Taugher et al., 2014). *Asic1a^−/−^* mice were maintained on a C57BL/6J background and *Asic1a^loxP/loxP^* mice were maintained on a C57BL/6T background. Male and female mice 12–18 weeks of age were used in the behavioral experiments and experimental groups were sex-matched and age-matched. All mice were kept on a 12 h light-dark cycle, with all experiments being performed during the light cycle. Mice were fed standard chow and water *ad libitum*. Animal care met the standards set by the National Institutes of Health and all experiments were approved by the University of Iowa Animal Care and Use Committee.

### Olfactory bulb lesions

Mice were anesthetized with a mixture of ketamine and xylazine. A burr hole was drilled above each olfactory bulb, and a glass pipette attached to vacuum suction was inserted into the burr hole to aspirate the olfactory bulb. Mice were sutured, and allowed to recover for at least 5 days before undergoing further experimentation. Sham surgeries, were performed as described above, but without insertion of the glass pipette. Ablation of the olfactory bulb was visually confirmed post-mortem.

### TMT-evoked freezing

Mice were placed in a clear, airtight plexiglas behavior chamber (20 × 21 × 17 cm, width × depth × height) with a beaker containing tissue paper carrying 6 μL undiluted trimethylthiazoline (TMT) (Contech enterprises) or 21 μL undiluted butyric acid (Sigma) applied with a micropipette. TMT volume sufficient to cause robust freezing in wild-type mice was empirically determined and butyric acid dose was adjusted for its difference in volatility as previously described (Hotsenpiller and Williams, 1997). Mice were videotaped, and freezing over a 10 min period was scored by a trained observer blinded to experimental conditions. Freezing was defined as an absence of motion other than respiration.

### Plethysmography

The rate of breathing was measured using standard plethysmographic techniques similar to those that have been previously described (Taugher et al., 2014). Mice were placed in a plethysmography chamber with continuous gas flow (700 ml/min). The protocol consisted of >10 min of baseline in compressed air, followed by an exposure to 3 μL TMT or 10.5 μL butyric acid. Volumes of TMT and butyric acid were proportionally reduced due to the smaller volume of the plethsmography chamber. All data were acquired using custom-written Matlab software. All data segments ≥5 s in duration that did not contain sighs, sniffing, or movement artifacts were selected for analysis. At least 30 s of data were analyzed for each condition. Breath volume and thus minute ventilation (rate × volume) could not be accurately measured because of the inability to adequately clean the temperature and humidity sensors after predator odor exposure, thus only respiratory rate was quantified. Data are reported as the percentage change in respiratory rate, relative to respiratory rate in compressed air.

### Viral injections

Adeno-associated viruses (AAV) were injected into the BNST and targeting was confirmed as previously described (Taugher et al., 2014). Briefly, AAV2/1 viruses expressing ASIC1A, Cre recombinase, or eGFP under control of a CMV (University of Iowa Gene Transfer Vector Core) were injected into the BNST bilaterally. AAV-ASIC1A and AAV-Cre were coinjected with AAV-eGFP to aid with localization. Coordinates (relative to bregma) were: anteroposterior +0.4 mm, lateral ± 1.0 mm, ventral 4.3 mm from the pial surface. Behavioral testing was done at least 3 weeks after virus injection. After behavioral testing, injection sites were located. Hits were defined as having bilaterally transduced the anterior BNST both dorsal and ventral to the anterior commissure. Mice in which the injections did not hit the BNST bilaterally were excluded including five *Asic1a^loxP/loxP^* mice and three *Asic1a*^−/−^ mice.

### ASIC1A immunohistochemistry

ASIC1A immunohistochemistry was performed as previously described (Taugher et al., 2014). Briefly, 18 μm cryosections were postfixed in 4% paraformaldehyde and 4% sucrose in PBS, then permeablized with 0.25% Triton X-100, then blocked with 5% goat serum, then immunostained with primary antibody [polyclonal anti-ASIC1 antiserum (MTY19) diluted 1:500 (Wemmie et al., 2003)], followed by immunostaining with secondary antibody [goat-anti rabbit IgG coupled to Alexa-Fluor-568 diluted 1:500 (Invitrogen)]. Sections were imaged with a confocal microscope (Zeiss 710).

### Statistical analysis

All values are reported as mean ± SEM. A Student's *t*-test was used to assess significance between two groups. Welch's correction was applied when comparing two groups of significantly different variance. An ANOVA was used to assess significance between more than two groups. Planned contrast testing (Student's *t*-test) was performed within the context of the ANOVA, to test for differences between two groups. *p* < 0.05 was considered significant for all tests. All statistical analyses were performed using Prism software (GraphPad).

## Results

### TMT reduces breathing and evokes freezing

**Figure 1:**
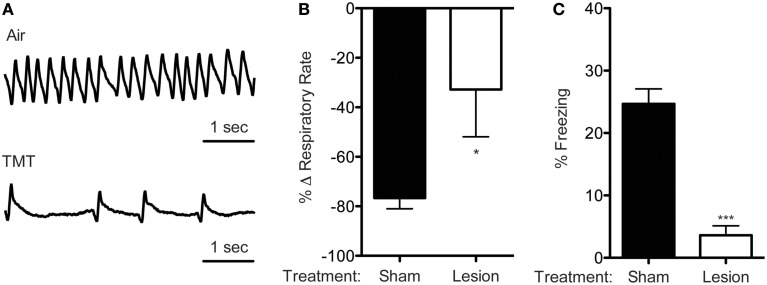
**TMT reduces breathing and evokes freezing. (A)** Representative plethysmography traces from a sham mouse in air and TMT. **(B)** TMT exposure dramatically reduces respiratory rate. This reduction in breathing rate is attenuated by olfactory bulb lesions [One-tailed *t*-test, *t*_(4)_ = 2.239, ^*^p = 0.0443, *n* = 5 per group]. **(C)** TMT also evoked freezing which was largely abolished by olfactory bulb lesions [*t*_(9)_ = 7.069, ^***^p < 0.0001, sham *n* = 6, lesion *n* = 5].

While observing mice during TMT exposure, we noticed that they appeared to breathe less frequently than during exposure to compressed air alone. Thus, we used whole-body plethysmography to quantify the effects of TMT on breathing rate, and indeed TMT exposure dramatically reduced rate of breathing (Figures 1A,B). In air, mice continuously took quick, rhythmic breaths, whereas in TMT, mice exhaled more slowly and there were distinct pauses between breaths (Figure 1A). Volume measurements were not possible with our plethysmographic set-up. Thus, it is not clear whether the reduction in breathing rate was accompanied by volume changes, although for each mouse it appeared that the amplitude of individual breaths did not change very much. This suggests an overall decrease in minute ventilation (rate × volume). Because a recent study in rats found that olfactory bulb lesions eliminated TMT-evoked freezing (Ayers et al., 2013), we wondered whether TMT effects on breathing rate might also depend on the olfactory bulb. Therefore, we lesioned the olfactory bulb bilaterally and tested breathing rate. We found that olfactory bulb lesions significantly attenuated effects of TMT on breathing rate (Figure 1B), as well as freezing (Figure 1C). Together, these observations are consistent with previous studies suggesting that effects of TMT depend on olfaction and extend those observations to another effect of TMT, reduction in breathing rate.

### Butyric acid reduces breathing rate but does not evoke freezing

**Figure 2:**
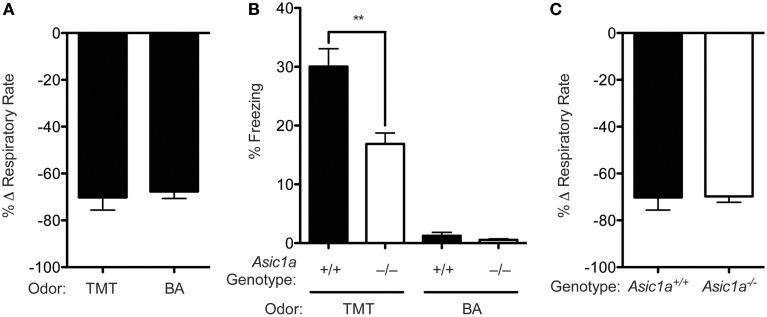
**Butyric acid reduces breathing rate but does not evoke freezing. (A)** Exposure to either TMT or butyric acid elicits a similar reduction in breathing rate in wild-type mice [*t*_(5)_ = 0.2768, *p* = 0.7930, TMT *n* = 5, butyric acid *n* = 2]. **(B)** Exposure to TMT, but not butyric acid (BA) elicits robust freezing behavior and *Asic1a* disruption attenuated TMT-evoked freezing. A Two-Way ANOVA revealed an effect of genotype [*F*_(1, 34)_ = 13.39, *p* = 0.0009, from left to right *n* = 10, 10, 9, 9, respectively], an effect of odor [*F*_(1, 34)_ = 141.7, *p* < 0.0001] and a genotype by odor interaction [*F*_(1, 34)_ = 10.78, *p* = 0.0024]. Planned contrast testing demonstrated that *Asic1a^−/−^* mice had a reduction in freezing to TMT (^**^p = 0.0016), but not butyric acid (BA) (*p* = 0.2673). **(C)** *Asic1a* disruption did not alter the effect of TMT on breathing rate [*t*_(8)_ = 0.08014, *p* = 0.9381, *n* = 5 per group].

We next sought to determine if the reduction in breathing rate observed in response to TMT might also be caused by other foul odors. Therefore, we tested the effects of butyric acid, another pungent odor, which, like TMT, is aversive and induces avoidance behaviors in rats and mice (Endres and Fendt, 2009). Similar to TMT, butyric acid dramatically reduced breathing rate (Figure 2A), suggesting the effect on breathing rate generalizes to at least two aversive odors. However, in contrast to TMT, butyric acid failed to induce freezing (Figure 2B), which is consistent with previous assertions that butyric acid differs from TMT in this regard (Coryell et al., 2007; Endres and Fendt, 2009). Thus, although both aversive odors caused mice to slow their breathing rate by a similar degree, the reduction in breathing rate by itself is probably not sufficient to cause the freezing behavior seen with TMT.

In previous studies, we found that acid sensing ion channels (ASICs) contribute to TMT-evoked freezing (Coryell et al., 2007, 2008; Price et al., 2014), and studies herein replicated the effect; in response to TMT, *Asic1a^−/−^* mice froze significantly less than *Asic1a^+/+^* controls (Figure 2B). Therefore, we wondered whether the breathing response to TMT might also be affected by ASIC1A. We repeated whole-body plethysmography with the *Asic1a^−/−^* mice and found that they were equally sensitive to the breathing rate reduction induced by TMT as *Asic1a^+/+^* controls. These results suggest that the *Asic1a^−/−^* mice were able to detect TMT normally. In addition, because TMT-induced breathing effects depended on olfaction, these results further suggest that the ability to smell TMT was intact in the *Asic1a^−/−^* mice (Coryell et al., 2007) (Figure 2C). Finally, these results suggest that the freezing deficit in the *Asic1a^−/−^* mice must be due to some other mechanism besides a deficit in olfaction.

### ASIC1A in the BNST is necessary for normal TMT-evoked freezing

**Figure 3:**
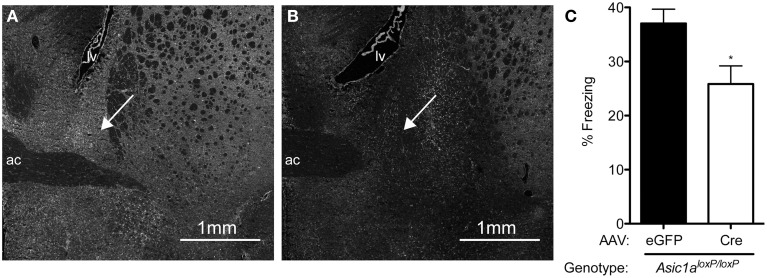
**ASIC1A in the BNST is necessary for normal TMT-evoked freezing. (A,B)** Examples of ASIC1A immunohistochemistry in the BNST of *Asic1a^loxP/loxP^* mice injected with AAV-eGFP or AAV-Cre, showing a decrease in ASIC1A expression in the BNST (arrow) in the AAV-Cre injected mouse. **(C)** Cre-mediated disruption of *Asic1a* in the BNST reduced TMT-evoked freezing [*t*_(30)_ = 2.643, ^*^p = 0.0129, AAV-eGFP *n* = 19, AAV-Cre *n* = 13].

ASIC1A is relatively abundant in many of the fear circuit structures that have been previously implicated in TMT-evoked freezing, including the BNST, PAG, and medial and basolateral amygdala (Wemmie et al., 2003; Coryell et al., 2007; Price et al., 2014). Because the BNST has been critically implicated in TMT-evoked freezing (Fendt et al., 2003), and because the BNST is a key site of ASIC1A action in freezing to another unconditioned stimulus, CO_2_ (Taugher et al., 2014), we hypothesized that the BNST might be a key site of ASIC1A action in TMT-evoked freezing. To test this hypothesis, we used AAV-Cre to selectively disrupt ASIC1A in the BNST of *Asic1a^loxP/loxP^* mice. Consistent with our previous observations (Taugher et al., 2014), injection of AAV-Cre into the BNST of *Asic1a^loxP/loxP^* mice effectively reduced ASIC1A expression the BNST, including both dorsal and ventral portions of the BNST and potentially in closely adjacent structures (Figures 3A,B). Next, we tested the behavioral effects of this manipulation on TMT-evoked freezing behavior. Disruption of ASIC1A in the BNST significantly reduced TMT-evoked freezing (Figure 3C), suggesting that the BNST is a key site of ASIC1A action in TMT-evoked freezing.

### ASIC1A in the BNST is sufficient to rescue TMT-evoked freezing deficits in Asic1a^−/−^ mice

**Figure 4:**
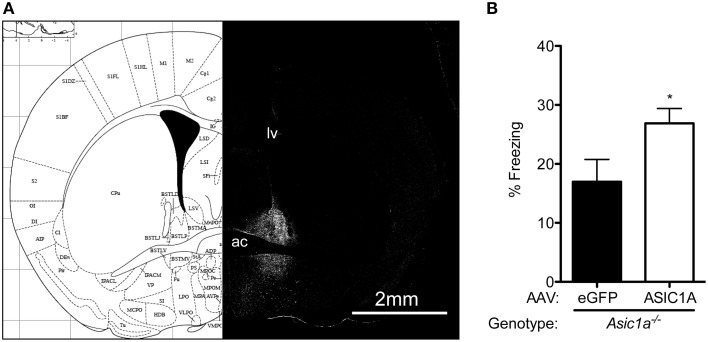
**Expression of ASIC1A in the BNST increases TMT-evoked freezing in *Asic1a^−/−^* mice. (A)** ASIC1A immunohistochemistry showing localized expression of ASIC1A in the BNST of *Asic1a^−/−^* mice injected with AAV-ASIC1A. For reference, the lateral ventricle (lv) and anterior commissure (ac) are labeled and a mirror image atlas schematic of the corresponding coronal section (Paxinos and Franklin, 2001) is shown. **(B)** Expression of AAV-ASIC1A in the BNST of *Asic1a^−/−^* mice increased TMT-evoked freezing [*t*_(12)_ = 2.249, ^*^p = 0.0441, AAV-eGFP *n* = 5, AAV-ASIC1A *n* = 9].

Because the previous data suggest that ASIC1A in the BNST is required for intact TMT-evoked freezing, we sought to test whether it might also be sufficient to increase TMT-evoked freezing in *Asic1a^−/−^* mice. To test this, we used an AAV to specifically express ASIC1A in the BNST of *Asic1a^−/−^* mice. Consistent with our previous observations (Taugher et al., 2014), we found that AAV-ASIC1A produced ASIC1A expression in both dorsal and ventral portions of the BNST (Figure 4A). Moreover, AAV-ASIC injected *Asic1a^−/−^* mice froze more to TMT than their AAV-eGFP injected counterparts (Figure 4B), suggesting that region-restricted expression of ASIC1A in the BNST is sufficient to augment TMT-evoked freezing.

## Discussion

During these studies, we observed that TMT can exert a striking reduction in breathing rate. TMT exposure caused mice to reduce breathing rate by up to 70% compared to compressed air. This likely translated to a reduction in minute ventilation, although mice remained awake and active for the duration of the TMT exposure, suggesting that ventilation was sufficient to maintain consciousness. In addition, if minute ventilation fell sufficiently, then one might expect a rise in systemic CO_2_ concentration and, in turn, an increase in breathing rate and/or volume through the hypercapnic ventilatory response (Hodges et al., 2008). However, for the duration of the TMT exposure, up to 10 min, we never observed a compensatory increase in breathing rate. However, there were periods of locomotor activity that interfered with the plethysmographic traces, during which time mice may have increased breathing rate and/or volume to compensate for the recorded bradypnea.

Why might mice slow their breathing in response to odors such as TMT and butyric acid? Interestingly, a previous study investigating anxiety-associated respiratory dysfunction in a mouse model of Rett syndrome reported respiratory irregularities during stressful stimuli which were attenuated by antalarmin, an antagonist of the corticotropin releasing hormone receptor 1 (Ren et al., 2012). In that study, TMT was one of the stressors tested and it was reported to induce apneic episodes, which were particularly striking in mice lacking MeCP2. Thus, one possible explanation is psychological stress, which conceivably might be induced by odors that are unpleasant or fear evoking. Alternatively, perhaps the breathing response is an effort to avoid the unpleasant smell or sense of disgust (Boiten, 1998), which might be volitional or avolitional. Acrid odors can often accompany dangerous substances; detecting and avoiding these odors can be critical for survival (Santos et al., 2004). Thus, it could be adaptive for an animal to reduce its rate of breathing until the smell has dissipated or the threat has passed.

In this study, olfactory bulb lesions attenuated the effect of TMT on breathing, suggesting that a large part of the TMT response is mediated through olfaction. However, some effect on breathing rate remained in the olfactory bulb lesioned mice. Although the lesions were confirmed to cause substantial damage, it is difficult to be certain that the olfactory bulb tissue was removed entirely. Alternatively, mechanisms besides olfaction might play a role. TMT might evoke a physiologic response through an unpleasant taste. Or, TMT might irritate mucosal tissues such as the eyes or airway, for instance through bitter taste receptors in lung mucosa (Shah et al., 2009). Additionally, it is conceivable that a compound as volatile as TMT might be absorbed systemically which might exert a variety of potential effects.

These studies highlighted two effects of TMT, slowed breathing rate and freezing. Previous studies have debated whether TMT induces its effects because of its repugnant odor or its association with a predator (McGregor et al., 2002; Blanchard et al., 2003; Fendt and Endres, 2008). Our data suggest that the breathing effect of TMT might be related to its repugnant odor. Another repugnant odor, butyric acid, also reduced breathing rate and by a magnitude similar to that induced by TMT. However, butyric acid did not induce freezing, indicating that the reduction in breathing rate alone was not sufficient to cause freezing and that the freezing effect is probably independent from the breathing effect. Furthermore, *Asic1a^−/−^* mice which froze significantly less in response to TMT exhibited a robust suppression of breathing that was equivalent to that observed in wild-type mice, suggesting that breathing, and freezing responses can be separable. Although the breathing response to TMT may be distinct from the fear-related freezing effects of TMT, understanding the underlying mechanisms may provide important insight into voluntary or involuntary control of breathing.

Our original objective was to investigate the potential sites of ASIC1A action in TMT-evoked behavior. Supporting our hypothesis that the BNST might be a critical site of ASIC1A action, we found that site-specific disruption of *Asic1a* in the BNST reduced freezing to TMT. Furthermore, viral-mediated restoration of ASIC1A expression in the BNST was sufficient to increase TMT-evoked freezing in *Asic1a^−/−^* mice. Although these observations strongly implicate the BNST, they do not rule out the possibility that ASIC1A in other brain regions might also play a role. Global *Asic1a* disruption altered TMT-evoked c-Fos activity in medial amygdala and periaqueductal gray, two structures that abundantly express ASIC1A (Coryell et al., 2007). ASIC1A might act at several sites in the circuit underlying TMT-evoked freezing, or loss of ASIC1A in the BNST may lead to changes in c-Fos activity elsewhere in the circuit. In addition, these studies raise questions about which BNST subnuclei are important for TMT-evoked freezing. Our viral manipulations altered ASIC1A in several BNST subnuclei, including the anterodorsal, and oval nuclei, which are thought to play opposing roles in anxiety-related behaviors (Kim et al., 2013). Further studies will be necessary to identify the key sites of ASIC1A action within the BNST and to evaluate other candidate sites of ASIC1A action in the TMT response.

A key question remains, how is this proton-gated channel ASIC1A involved in TMT-evoked freezing? One possible answer is that TMT might induce a systemic acidosis by reducing breathing rate and thus raising CO_2_ levels. This seems to be an unlikely explanation, however, as butyric acid had a similar effect on breathing, but did not elicit freezing. Moreover, we did not observe an increase in respiratory rate, even after prolonged TMT exposure, which suggests that CO_2_ did not reach a level sufficient to trigger the hypercapnic ventilatory response (Corcoran et al., 2009). Alternatively, TMT might lower pH in the circuit by inducing synaptic transmission. Neurotransmitter containing vesicles are acidic and during vesicle release can lower pH in the synaptic cleft (Du et al., 2014). Perhaps the two potential mechanisms highlighted above act in combination whereby systemic acidosis potentiates localized changes in pH due to synaptic transmission or another source such as local metabolic activity. Conversely, although ASIC1A is a proton-gated channel, it might contribute to the TMT response in some way other than by directly detecting acidosis. Recent studies suggest that ASIC1A may influence synaptic structure in brain regions other than the BNST (Zha et al., 2006; Du et al., 2014; Kreple et al., 2014). Therefore, it is possible that as in these other structures ASIC1A in the BNST might alter dendritic spine number and morphology, AMPA/NMDA ratio, and/or synaptic currents.

Finally, the observation that ASIC1A in the BNST contributes to both TMT- and CO_2_-evoked freezing behaviors raises the possibility that ASIC1A and the BNST might contribute to other unconditioned fear-related responses in mice, and also possibly in humans. Unconditioned fear is thought to play a role in psychiatric diseases, particularly specific phobias, in which patients exhibit a strong unconditioned response to certain objects or situations (Kessler et al., 2005; Choy et al., 2007). ASIC1A in the BNST might also contribute to other anxiety disorders, as variations in the ASIC1A-encoding genomic locus have been recently associated with panic disorder (Smoller et al., 2014).

### Conflict of interest statement

The authors declare that the research was conducted in the absence of any commercial or financial relationships that could be construed as a potential conflict of interest.
